# Ametropia detection using a novel, compact wavefront autorefractor

**DOI:** 10.1111/opo.13263

**Published:** 2023-12-12

**Authors:** Carlos S. Hernández, Andrea Gil, Amal Zaytouny, Ignacio Casares, Jesús Poderoso, Alfonso de Lara, Alec Wehse, Shivang R. Dave, Daryl Lim, Eduardo Lage, Nicolas Alejandre-Alba

**Affiliations:** 1 Department of Electronics and Communications Technology, Universidad Autónoma de Madrid, Madrid, Spain; 2 PlenOptika, Inc., Boston, Massachusetts USA; 3 Instituto de Investigación Sanitaria de la Fundación Jiménez Diaz, Madrid, Spain; 4 Instituto de Óptica “Daza de Valdes”, Spanish National Research Council, CSIC, Madrid, Spain; 5 Ophthalmology Department, Fundación Jiménez Diaz Hospital, Madrid, Spain

**Keywords:** autorefractor validation, QuickSee Free, refractive errors, subjective refraction

## Abstract

**Introduction:**

Despite the well-known reproducibility issues of subjective refraction, most studies evaluating autorefractors compared differences between the device and subjective refraction. This work evaluated the performance of a novel handheld Hartmann–Shack-based autorefractor using an alternative protocol, which considered the inherent variability of subjective refraction.

**Methods:**

Participants underwent an initial measurement with a desktop autorefractor, two subjective refractions (SR1 and SR2) and a final measurement with the QuickSee Free (QSFree) portable autorefractor. Autorefractor performance was evaluated by comparing the differences between the QSFree and each of the subjective refractions with the difference between the subjective refractions (SR1 vs. SR2) using Bland–Altman analysis and percentage of agreement.

**Results:**

A total of 75 subjects (53 ± 14 years) were enrolled in the study. The average difference in the absolute spherical equivalent (*M*) between the QSFree and the SR1 and SR2 was ±0.24 and ±0.02 D, respectively, that is, very similar or smaller than the SR1 versus SR2 difference (±0.26 D). Average differences in astigmatic components were found to be negligible. The results demonstrate that differences between QSFree and both subjective refractions in *J*_0_ and *J*_45_ were within ±0.50 D for at least 96% of the measurements. The limits of agreement (LOAs) of the differences between QSFree and SR1, as well as QSFree and SR2, were higher than those observed between SR1 and SR2 for *M*, *J*_0_ and *J*_45_.

**Conclusions:**

A protocol was designed and validated for the evaluation of a refractive device to account for the variability of subjective refraction. This protocol was used to evaluate a novel portable autorefractor and observed a smaller difference between the device and subjective refractions than the difference between the two subjective refraction measurements in terms of mean bias error, although the standard deviation was higher.

**Supplementary Information:**

The online version of this article (doi:10.1111/opo.13263) contains supplementary material, which is available to authorized users.

## Key points

- Refractive prescriptions obtained from an autorefractor can be an effective tool for extending eyecare coverage to a broader population base, especially in low- and middle-income countries and remote areas where there is shortage of optometrists and facilities to deliver refractive care services.
- Most studies assessing the accuracy of autorefractors have used subjective findings obtained by an experienced optometrist for comparison. While this methodology is commonly accepted, it fails to consider the inherent variability of the subjective refraction.
- This paper proposes an alternative approach for assessing the accuracy of an autorefractor by comparing the difference between the autorefractor and two subjective findings versus the difference between the two subjective results.

## INTRODUCTION

Uncorrected refractive errors are the leading cause of moderate to severe vision impairment globally. It is estimated that at least 900 million people suffer from vision loss that could be addressed with an appropriate pair of spectacles; a number that is projected to increase due to the population growth in low- and middle-income countries, the longer lifespans and proportion of the elderly individuals in society and the accelerating myopia epidemic.^1,2^ Vision loss is also driven by the inequality in access to eyecare, with 90% of those with uncorrected refractive errors living in low- and middle-income countries^3^ where they face a shortage of trained eyecare professionals (ECPs), equipment and facilities to deliver refractive care services and optical dispensing units.^4^

In recent years, relatively inexpensive and portable autorefractors have been developed and commercialised in an attempt to alleviate the burden of uncorrected refractive errors.^5–8^ Such handheld technologies can be effective tools for extending eyecare coverage to a broader population base, especially in low- and middle-income countries and remote areas, or for wheelchair or bedbound patients. Additionally, the ease of use and high repeatability increase their suitability for deployment in large-scale refractive error screening and eyeglass delivery programmes.

The scientific literature has begun to provide evidence supporting the wider adoption of autorefractor-based refractive prescriptions as a practical and viable approach to address the global problem of uncorrected refractive errors.^7–11^ However, there is often a measurable difference between the autorefractor measurement and the clinical gold standard, namely subjective refraction. Such differences have been attributed to a range of factors, including the patient's accommodation, ambient light conditions, the specific autorefraction technology, severity and type of ametropia, patient's cognitive ability or the experience of the ECP.^12,13^ In this regard, Mathebula and Rubin^14^ investigated the reproducibility of the subjective refraction in a single symptomatic participant using the findings from 50 experienced optometrists and obtained reproducibility limits for the spherical equivalent of up to ~1.00 D. This finding is consistent with Mackenzie^12^ where reproducibility was evaluated in an asymptomatic single participant with 40 examiners performing subjective refractions. Although other differently designed studies (summarised by Mathebula and Rubin^14^) have suggested markedly better reproducibility of subjective refraction, the aggregated evidence makes it crucial to consider the inherent variability of the subjective refraction process when validating autorefraction technology.

The current study introduces and clinically evaluates a pre-commercial prototype of a novel handheld, open-view, monocular Hartmann–Shack-based autorefractor, named QuickSee Free (QSFree), manufactured by PlenOptika (plenoptika.com/quicksee-free), which incorporates an advanced version of a well-established wavefront refraction technology.^8,11,15–19^ The new design of the optical system and software algorithms allow for an extended myopic measurement range and the incorporation of a pupil camera within an ultra-compact device. QSFree, unlike its predecessor QuickSee, was designed so that the operator holds the device to the patient's face to improve usability while maintaining high levels of precision and accuracy. To validate this novel device, a new study design was proposed to account for the variability in the subjective refraction process. Specifically, in contrast to previous clinical investigations that have validated portable autorefraction technologies by evaluating the difference between the device and subjective refraction by a single ECP,^7,9,20^ this study compared the differences between the autorefractor and subjective refractions performed by two independent ECPs with the differences between the subjective refraction measurements (as performed by the two ECPs). This provided a measure of the device's accuracy against the inherent variability of the subjective refraction procedure.

## MATERIALS AND METHODS

### QuickSee Free prototype

The QSFree prototype tested here is a compact, open-view, monocular, operator-held wavefront autorefractor implementing simplified Shack–Hartmann architecture. The device is designed to measure each eye sequentially with spherical errors from −14 to +10 D and astigmatism in the range of −8 to +8 D. The weight of the device, including the internal battery (with 10 Ampere-hours [Ah] of electrical charge), is 746 g and its physical dimensions are 30 × 18 × 6 cm (height × length × width).

**FIGURE 1:**
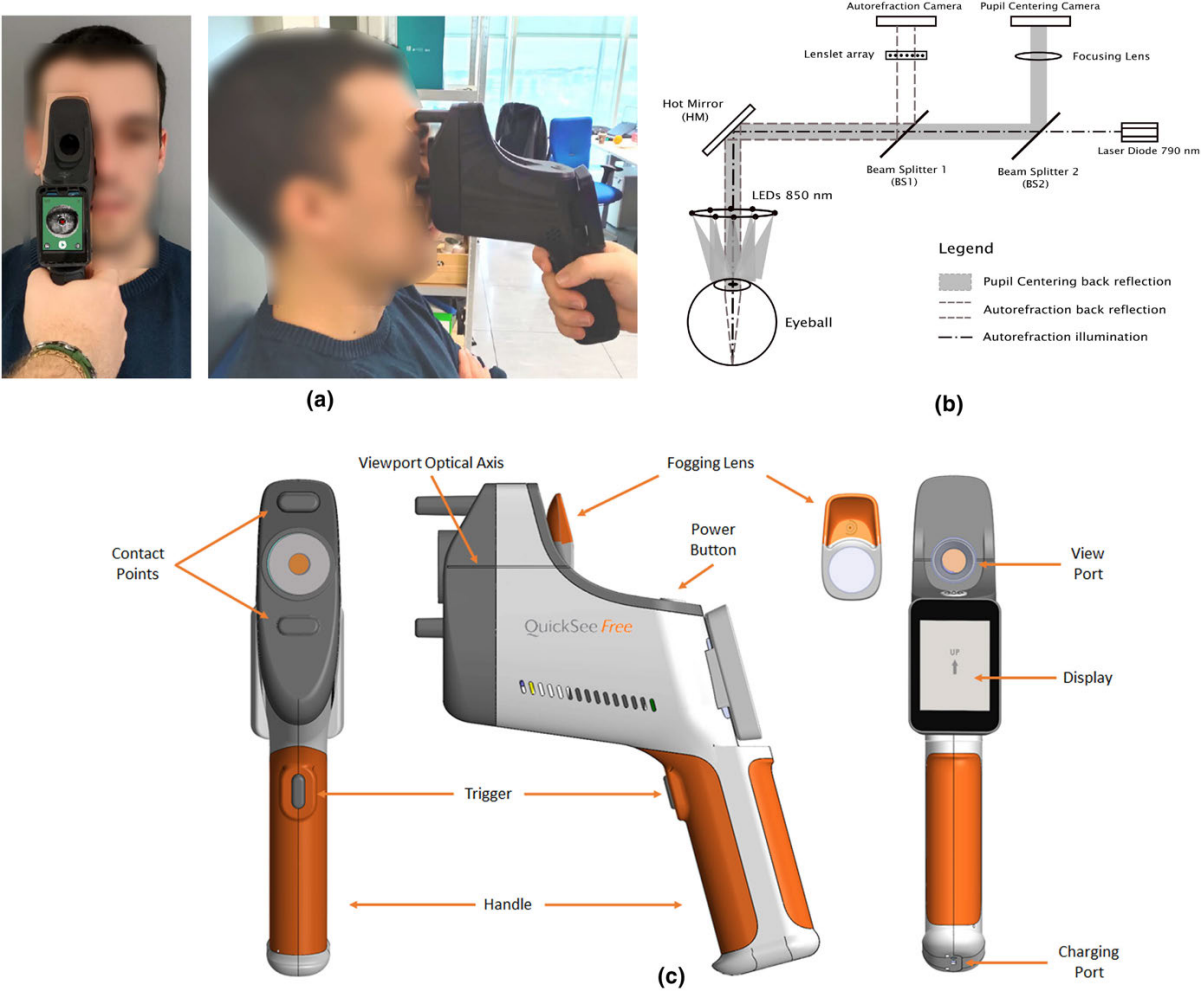
(a) Photographs of the QuickSee Free (QSFree) prototype in use. The operator places the device on the patient's face with one hand. The patient is instructed to look through the device at a distant VA chart while the contralateral eye is occluded (not shown in picture). (b) Layout of the optical components of the QSFree open-view Hartmann–Shack-based autorefractor. (c) Design of the QSFree. The device has two anchoring points, a pivoting touchscreen and a detachable fogging lens.

To perform a measurement, the operator aligns the viewport of the device with the eye by referencing the angle between the patient's optical axis and the horizontal line printed on the side of the viewport, which is co-planar with the optical axis of the viewport (Figure 1c). That position is held steady during the measurement by gently resting the device against the patient's face using two anchoring points equipped with soft rubber tips (Figure 1a,c). During the centring process, real-time pupil images are displayed on a built-in screen along with a reference marker (a red spot on the display, Figure 1a). The operator aligns this marker with the patient's pupil by making small adjustments to the position of the device, guided by the live pupil images. The user interface also provides visual feedback by turning green when alignment is achieved. Upon alignment, the acquisition process can be initiated by pressing the touchscreen or a trigger button on the instrument's handle.

A simplified diagram of the optical design of the device is shown in Figure 1b. The autorefractor module uses a 790-nm Class-I laser that creates a point source on the retina which is reflected back as a wavefront that aberrates as it propagates through the optical system of the eye. The wavefront is analysed with a custom sensor consisting of a microlens array and a 1.3-megapixel camera using complementary metal oxide semiconductor (CMOS) technology. The alignment module (pupil centring in Figure 1b) uses 850 nm LEDs for illumination, a lens for focusing the eye at the desired working distance and the same 1.3-megapixel CMOS camera employed in the autorefraction module. Further details about the technical performance and custom data processing algorithms have been described previously.^15–18^

The open-view design of the QSFree allows the subject to fixate on a distant target during the measurement. Numerous studies have demonstrated that an open-view design, when incorporated into an autorefractor, both reduce and control accommodation.^10,21^ Additionally, the device can be equipped with a magnetically detachable fogging lens positioned in the field of view of the eye being measured, thereby further facilitating the relaxation of accommodation^22,23^ (Figure 1c). Additionally, the QSFree can be used either with both eyes open or with the contralateral eye occluded during the measurement, depending on the operator's preference and other factors such as the environmental conditions.

### Study protocol

Participants were recruited from the Department of Ophthalmology of the Fundación Jiménez Díaz (FJD) hospital (Madrid, Spain), during routine visits. Inclusion criteria were (1) no history of surgery or eye disease, (2) between 18 and 75 years of age, (3) corrected visual acuity (VA) of 6/7.5 or better in each eye, (4) no use of systemic or ocular medication that may affect vision and (5) refractive error in the range of −14 to +10 D (spherical equivalent) and astigmatism ≤+8 D. The research was approved by the hospital's institutional review board and followed the tenets of the Declaration of Helsinki. The protocol was also approved by the Spanish Agency of Medicines and Medical Products (AEMPS), with file number 991/22/CE-R.

Participants followed standard refraction procedures consisting of an objective assessment of refractive error using a single measurement from a desktop autorefractor (Topcon KR-8900, topconhealthcare.com/article/product-category/auto-refractometer),^24,25^ followed by subjective refinement (SR1) performed by a clinical optometrist. Patients who completed this procedure and met the eligibility criteria were invited to participate in the study.

Enrolled subjects were asked to read and sign the informed consent form and were escorted to an alternative examination room where a second optometrist, dedicated exclusively to this study, performed another comprehensive subjective refraction (SR2) using the same measurement of the desktop autorefractor as the starting point, with the same equipment and protocol as for the SR1. The second optometrist was masked to the SR1 results to avoid possible bias.

Finally, during the first QSFree measurement, participants were instructed to look through the device at the 6/30 line of a VA chart positioned at a distance of 6 metres, while they held a standard occluder in front of the contralateral eye. In order to determine the intrasession repeatability of the device, the same optometrist performed a second measurement with the QSFree in a subgroup of 21 patients, with the measurements being spaced 3 minutes apart. To mitigate the effects of accommodation, all measurements were taken with a +2.00 D fogging lens attached to the device^22,26^ (Figure 1c). This protocol was selected to minimise potential sources of uncertainty in the measurements (use of a fogging lens to reduce accommodation and occlusion of the contralateral eye to minimise peripheral distractions) while providing easy and reproducible instructions.

Binocular and monocular VAs achieved with the SR1 and SR2 were recorded and included in the analysis to assess the variability of the subjective refraction procedure. Sample size calculations were conducted with the goal of detecting differences in spherical equivalent refractive error of ±0.25 D, with a minimum power of 80% (*α* = 0.05). The mean difference in spherical equivalent between subjective refraction and QSFree autorefraction was assumed to be −0.09 ± 0.79 D, based on findings from several previous studies.^15^ Considering these assumptions and using a bilateral equality test, it was determined that a total sample size of 72 subjects was required. Therefore, to account for any dropouts during the study, a goal of recruiting at least 100 subjects was established.

### Data analysis

Statistical analysis was performed in the power vector domain.^27^ The measurements obtained from the right eye from the SR1, SR2 and QSFree were converted to their equivalent power vector parameters of spherical equivalent (*M*), vertical Jackson's cross cylinder (*J*_0_) and oblique Jackson's cross cylinder (*J*_45_).

Statistical methods employed to compare the differences: SR1-SR2, SR1-QSFree and SR2-QSFree refractions were (1) two-way mixed effect intraclass correlation coefficient (ICC) from a single measurement,^28^ to determine if a linear relationships existed between the different refraction components; (2) Bland–Altman analysis, since this is the standard procedure when evaluating the accuracy and precision of two separate methodologies^29^ and (3) the percentage of agreement between prescriptions within ±0.25 and ±0.50 D classified by refractive error type (myopic, hyperopic or emmetropic). Furthermore, double-angle astigmatic plots were used to better visualise differences in the cylindrical components.

To ensure that the analysis of agreement was not biased by the fact that SR1 was performed by multiple optometrists, a rigorous statistical analysis was performed using the Wilcoxon rank-sum test since the data were not normally distributed. The medians of the differences SR1–SR2, SR1–QSFree and SR2–QSFree were compared before and after random removal of the participants for a single optometrist. Thus, there were as many comparisons as optometrists in the SR1 group. In each comparison, subjects examined by one of the optometrists were excluded from the data set.

Intrasession repeatability was assessed using Bland–Altman analysis for repeated measurements obtained from the subgroup of subjects. The average agreement between the two measurements was determined by calculating the mean inter-patient difference, while repeatability was evaluated using the 95% limits of agreement (LOAs) of these differences (calculated as 1.96 times the standard deviation).

All VA measurements were converted into the logarithm of the minimum angle of resolution (LogMAR) for statistical comparison. Monocular and binocular VAs from SR1 and SR2 were compared using a histogram of the difference in VA achieved by each participant.

## RESULTS

A total of 75 adults with ages ranging from 20 to 74 years were recruited for the study. The mean ± SD age of the participants was 53 ± 14 years. The initial subjective refraction (SR1) was performed by five hospital optometrists, who refracted 38.6%, 16%, 12%, 26.6% and 6.8% of the patients, respectively. The second subjective refraction (SR2) and measurements with the QSFree were all performed by a single optometrist dedicated exclusively to the study. Table 1 presents the number of subjects per refractive group as determined by SR1, SR2 and the QSFree autorefractor. Although the proportions found in the SR1 and SR2 were similar and the examinations were performed under similar conditions, there were some minor discrepancies due to the aforementioned variability of the subjective refraction procedure.^14^

**TABLE 1:** Number of subjects in each refractive error range according to the right eye prescriptions provided by the first (SR1) and second (SR2) subjective refraction and the QSFree device.

Condition	# Patients SR1 (%)	# Patients SR2 (%)	# Patients QSFree (%)
Emmetropes			
−0.50 D < *M* < +0.50 D	14 (18.67)	16 (21.33)	19 (25.33)
Myopes			
−3.50 D < *M* ≤ −0.50 D	13 (17.33)	15 (20.00)	11 (14.67)
*M* ≤ −3.50 D	11 (14.67)	11 (14.67)	12 (16.00)
Hyperopes			
+0.50 D ≤ *M* < +2.00 D	21 (28.00)	19 (25.33)	23 (30.67)
+2.00 D ≤ *M*	16 (21.33)	14 (18.67)	10 (13.33)

Abbreviation: *M*, spherical equivalent refractive error.

Table 2 shows the refractive error distribution for the three power vector components (*M*, *J*_0_ and *J*_45_) based on the refractions from SR1, SR2 and QSFree. Based on the manifest refraction data, the uncorrected spherical equivalent for SR1 and SR2 ranged from −11.50 to +5.50 D and from −12.38 to +5.13 D, respectively.

**TABLE 2:** Mean, standard deviation, minimum and maximum values for the *M*, *J*_0_ and *J*_45_ components for the first (SR1) and second (SR2) subjective refraction and the QSFree device.

	SR1	SR2	QSFree
*M*			
Mean *M* (D)	−0.20	−0.45	−0.44
SD *M* (D)	+3.15	+3.18	+3.15
Min *M* (D)	−11.50	−12.38	−12.00
Max *M* (D)	+5.50	+5.13	+4.50
*J*_0_			
Mean *J*_0_ (D)	+0.00	−0.01	−0.05
SD *J*_0_ (D)	+0.48	+0.47	+0.48
Min *J*_0_ (D)	−1.25	−1.25	−1.41
Max *J*_0_ (D)	+1.76	+1.75	+1.35
*J*_45_			
Mean *J*_45_ (D)	−0.05	−0.01	0.04
SD *J*_45_ (D)	+0.26	+0.24	+0.27
Min *J*_45_ (D)	−1.47	−1.23	−1.06
Max *J*_45_ (D)	+0.40	+0.62	+0.76

**FIGURE 2:**
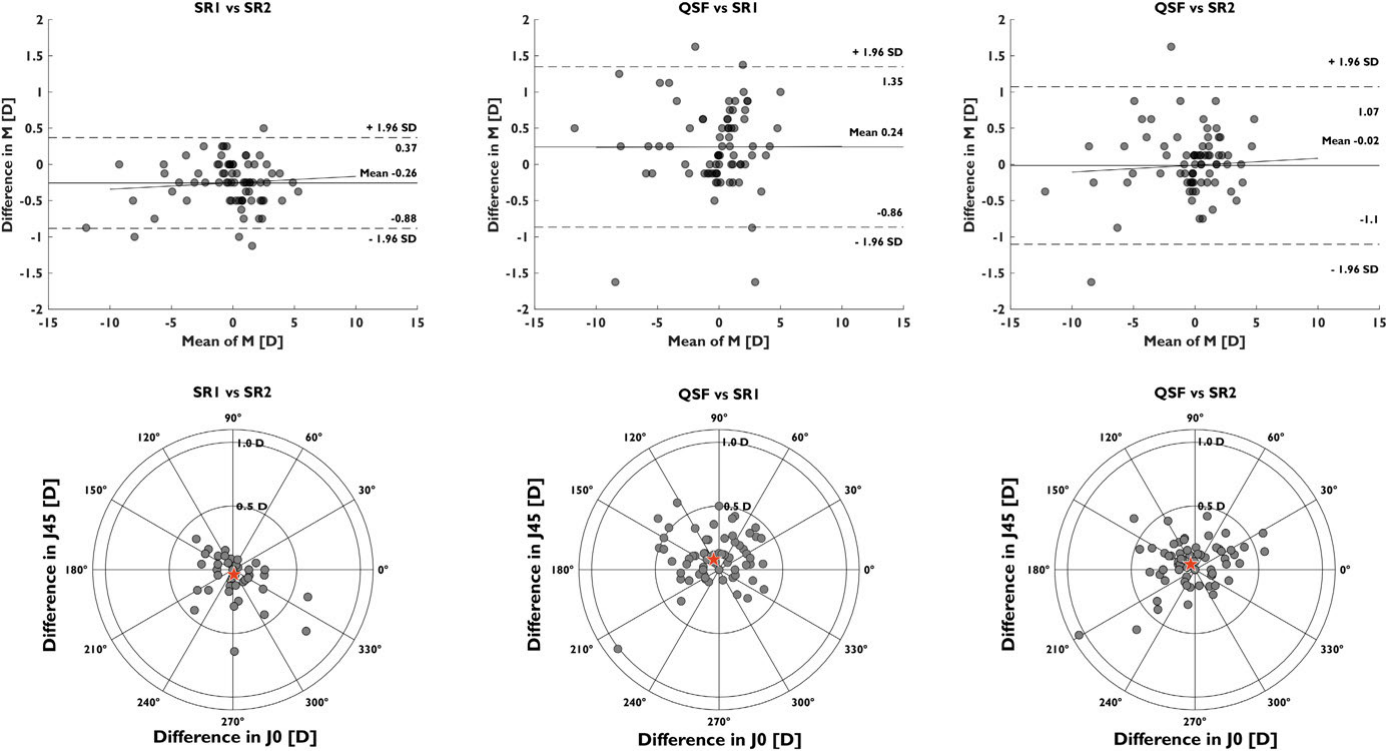
Top: Bland–Altman plots comparing the agreement in spherical equivalent (*M*) between the three methods. Bottom: double-angle astigmatic plots showing the agreement of *J*_0_ and *J*_45_ for the three methods. The stars shown in the double-angle astigmatic plots represent the centre of the distribution found in each case. SR1, first subjective refraction; SR2, second subjective refraction.

Table 3 and Figure 2 present a summary of the ICC and Bland–Altman analysis. High correlation coefficients were found between the measurements obtained with the different methods, especially for the *M* value. The mean bias for *M* between QSFree and SR2 (−0.02 D) was, in absolute terms, significantly smaller than the bias between SR1 and SR2 (−0.26 D), as well as between QSFree and SR1 (+0.24 D). The mean bias error for *J*_0_ and *J*_45_ showed similar results for all three methods. The 95% LOA between the QSFree and either of the two subjective refractions was higher than the LOA between SR1 and SR2. Due to the non-normal distribution of the refractive errors in the study, we conducted significance tests for the paired mean differences using the Wilcoxon signed-rank test. Across all cases examined, no statistically significant differences were found (*p*- > 0.05) in the error distribution of the power vector components measured between the three methods.

**TABLE 3:** Intraclass correlation coefficients (ICCs), mean bias, 95% limits of agreement (LoAs) and *p*-values (*p*) obtained by Wilcoxon sign-rank test for the different refraction methods for all power vector components. SR1, first subjective refraction; SR2, second subjective refraction.

Comparison	*M*				*J*_0_				*J*_45_			
	ICC	Mean bias	95% LoA	*p*	ICC	Mean bias	95% LoA	*p*	ICC	Mean bias	95% LoA	*p*
SR1 vs. SR2	0.99	−0.26	±0.63	0.47	0.95	−0.01	±0.29	0.49	0.84	+0.04	±0.26	0.17
SR1 vs. QSFree	0.98	+0.24	±1.11	0.56	0.89	+0.04	±0.44	0.74	0.72	−0.08	±0.36	0.08
SR2 vs. QSFree	0.98	−0.02	±1.09	0.88	0.87	+0.03	±0.38	0.75	0.77	−0.04	±0.33	0.18

Percentages of agreement between SR1, SR2 and QSFree grouped in terms of myopes, hyperopes and emmetropes (based on the Topcon KR-8900 refractions), as well as for the entire population, are shown in Table 4. For emmetropes, the percentages of agreement were very high between the three methods. Nearly 100% of the *M*, *J*_0_ and *J*_45_ values lay within the ±0.50 D range, except for SR2 which showed 93.33% and 92.31% agreement with SR1 and QSFree, respectively, for the *J*_0_ component. In the hyperopic group, the QSFree showed a higher agreement with SR2 (80.55% [*M*], 97.22% [*J*_0_] and 97.22% [*J*_45_]) than with SR1 (58.33% [*M*], 97.22% [*J*_0_] and 97.22% [*J*_45_]) for the 0.50 D threshold, but was in line with the agreement between SR1 and SR2 for the same threshold (83.33% [*M*], 97.22% [*J*_0_] and 100% [*J*_45_]). For myopes, the agreement of QSFree with SR1 and SR2 for *J*_0_ and *J*_45_ was 100% but lower for *M*, being 65.21% and 69.56%, for SR1 and SR2, respectively. Overall, the percentage of measurements within the ±0.50 D range between QSFree and SR2 (81.33% [*M*], 96.00% [*J*_0_], 98.67% [*J*_45_]) were very similar to those between SR1 and SR2 (88.00% [*M*], 97.33% [*J*_0_], 98.67% [*J*_45_]) for the same range.

**TABLE 4:** Agreement between the different refractions methods showing the percentage of measurement that fell within ±0.25, ±0.50 and ±1.00 D for the *M*, and ±0.25, ±0.50 D for *J*_0_ and *J*_45_. SR1, first subjective refraction; SR2, second subjective refraction.

	*M* agreement within			*J*_0_ agreement within		*J*_45_ agreement within	
	±0.25 D	±0.50 D	±1 D	±0.25 D	±0.50 D	±0.25 D	±0.50 D
Emmetropes							
SR1 vs. SR2	80.00%	100%	100%	93.33%	93.33%	93.33%	100%
QSFree vs. SR1	80.00%	100%	100%	80.00%	100%	86.67%	100%
QSFree vs. SR2	73.33%	100%	100%	73.33%	92.31%	86.67%	100%
Hyperopes							
SR1 vs. SR2	47.22%	83.33%	97.22%	94.44%	97.22%	94.44%	100%
QSFree vs. SR1	38.89%	58.33%	94.44%	75.00%	97.22%	80.55%	94.44%
QSFree vs. SR2	61.11%	80.55%	97.22%	80.56%	97.22%	86.11%	97.22%
Myopes							
SR1 vs. SR2	73.91%	86.97%	100%	91.30%	100%	91.30%	95.65%
QSFree vs. SR1	56.52%	65.21%	78.26%	73.91%	100%	82.61%	100%
QSFree vs. SR2	56.52%	69.56%	94.44%	78.26%	100%	86.96%	100%
Entire population							
SR1 vs. SR2	62.67%	88.00%	98.67%	93.33%	97.33%	93.33%	98.67%
QSFree vs. SR1	53.33%	69.33%	90.67%	76.00%	98.67%	82.67%	97.33%
QSFree vs. SR2	62.67%	81.33%	96.00%	78.67%	96.00%	86.67%	98.67%

The data presented in Table S1 show that the agreement analysis was not biased by any of the five optometrists who performed SR1. The results of the Wilcoxon rank-sum test show *p*-values ≥ 0.25 for comparisons of the three power vectors. These results indicate that when randomly excluding patients from any of the five practitioners, no significant differences were found in the medians and LOA of the error distributions for the remaining patients.

Bland–Altman plots illustrating intrasession repeatability of the QSFree in 21 subjects are shown in Figure S1. For all three power vector components, the device was very consistent, with average differences for *M*, *J*_0_ and *J*_45_ of +0.01, 0.00 and −0.01 D, respectively, and 95% LOA of ±0.46, ±0.18 and ±0.19 D, respectively.

**FIGURE 3:**
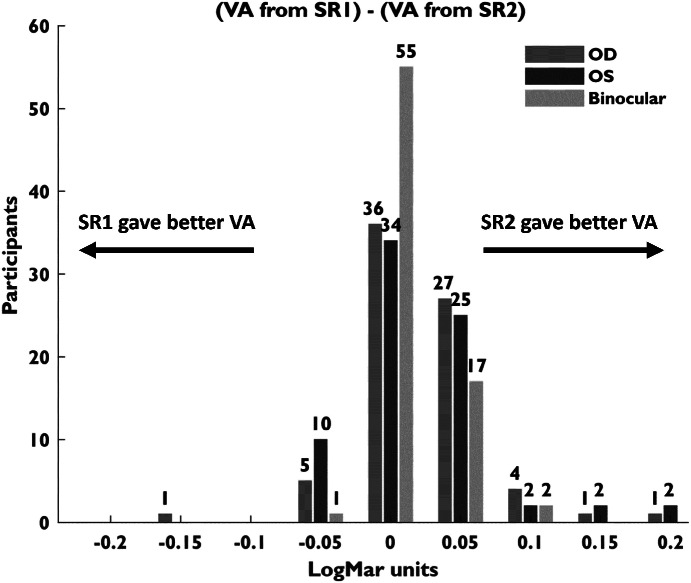
Differences in visual acuity (VAs) measured at the two subjective examinations (SR1 and SR2). OD, right eye; OS, left eye.

Figure 3 shows the differences in VAs (binocular and monocular) for SR1 and SR2. Binocular VA based on the SR2 prescriptions (−0.09 ± 0.02 LogMAR, mean ± SD) were better, equivalent or worse than those obtained from SR1 (−0.08 ± 0.03 LogMAR, mean ± SD) in 25.33%, 73.33% and 1.33% of the patients, respectively. Despite these differences in VAs, participants reported being comfortable with either finding.

## DISCUSSION

Most studies assessing the accuracy of autorefractors compare the objective findings with subjective refraction performed by a single experienced optometrist. While this approach is widely accepted, it does not account for the inherent variability of the subjective procedure.^12–14,30,31^ In this work, we proposed an alternative study design that considered this variability by comparing the difference between the autorefractor readings and two subjective refractions performed by different optometrists with the difference between the two subjective examinations.

Intraclass correlation coefficient analysis (Table 3) obtained correlation coefficients approaching unity for the *M* value between the three methods (SR1, SR2 and QSFree). The astigmatic correlations were lower than those observed for the *M* component in all cases, especially for the *J*_45_. In particular, the *J*_0_ and *J*_45_ ICC coefficients between the QSFree and SR1 (0.89 and 0.72, respectively) and between the QSFree and SR2 (0.88, 0.77, respectively) were lower than the correlation between SR1 and SR2 (0.95, 0.85, respectively). This difference in astigmatism may be explained by misalignment (roll, pitch and yaw) when the device is placed on the subject's face (Figure 1a) and the fact that optometrists sometimes prefer (standard clinical practice) to undercorrect the astigmatism (usually up to ~0.50 D) for increased comfort and acceptance by the patient. Despite this reduced ICC between the QSFree and the subjective refraction compared with the value between SR1 and SR2 for astigmatism, the distribution of differences shown in the double-angle astigmatic plots (Figure 2, bottom) was very similar in all cases, with negligible bias and most differences lying within ±0.50 D.

Comparison of the percentage of patients with refractions within ±0.25 and ±0.50 D for the three methods showed that the performance of the QSFree was closer to SR2 than to SR1 (Table 4). The agreement for astigmatism was also very high between all methods. Adopting the ±0.50 D range, then almost 100% of the *J*_0_ and *J*_45_ measurements agreed for all refractive groups. For the *M* values, the comparison of agreement showed variability between the different refractive groups. For emmetropes, the percentage of agreement was high, with 100% of the *M* measurements falling within the ±0.50 D range. For hyperopes, the highest agreement within the ±0.50 D range was between the subjective refractions, that is, 83.33% for *M*, followed closely by the agreement between the QSFree and SR2 (80.55% for *M*). These results may be related to the use of a fogging lens to relax accommodation. Previous studies have shown that the use of fogging lenses may result in a similar reduction in accommodation to the use of cycloplegia in emmetropic and hyperopic subjects, while being less invasive.^22^ Overall, the lowest agreement between the device and subjective refractions for *M* was found in the myopic group. Using the ±0.50 D threshold, the agreement between SR1 and SR2 was 88.00%, while the agreement between QSFree and SR2 was 69.56%, although this increased to 94.44% with a ±1.0 D range. These differences can be explained by the relatively small pupil diameters (3.0 mm) recorded by the QSFree prototype here. It was observed that excessive ambient light entering through the viewport of the device decreased the pupil diameter and compromised the quality of the Hartmann–Shack-based images in some subjects. Although this issue has been resolved in the commercial version of the device by modifying the visible light filter in the measurement channel, it is possible that the findings of this study in the myopic group were impacted by these factors, thereby masking the true performance of the device in these participants. An additional factor that could have contributed to the reduced accuracy in the myopic group was the use of the fogging lens. This may have led to some individuals with mild myopia to experience discomfort while looking through the device and not focusing on the internal target, thereby inducing accommodation during the measurement.

In this investigation, children were excluded due to their greater accommodative ability compared with adults. Although a wide age range of the participants was seen (20–74 years), the average age of the sample was 53 ± 14 years, due to inherent patient demographics at the study site. A consequence of the population distribution is that accommodation would have less influence on the overall results.

Bland–Altman analysis (Table 3 and Figure 2) determined a smaller bias in *M*, in terms of absolute values, between QSFree and SR2 (−0.02 D) than either SR1 and SR2 (−0.26 D) or QSFree and SR1 (+0.24 D). The LOA between SR1 and SR2 for *M* (±0.63 D) were lower than between QSFree and SR1 or SR2 (±1.09 and ±1.11 D, respectively). This may be related to the fact that the same autorefraction reading was used as the starting point for both SR1 and SR2. The variability in subjective refraction for *M* observed here was slightly lower than that found in previous studies quantifying this parameter,^12,14^ in which 40–50 subjective refractions were used for the estimation. The LOA reported in earlier investigations (±1.0 D) was comparable with the value noted here between QSFree and subjective refraction and in other recent works validating portable autorefractors.^7,11,15,20^ In contrast, the LOAs and mean bias error for the astigmatic components (*J*_0_, *J*_45_) were comparable between the three methods. Compared to other portable autorefractors, LOAs between QSFree and SR1 (±0.44 D [*J*_0_], ±0.36 D [*J*_45_]) and between QSFree and SR2 (±0.38 D [*J*_0_], ±0.33 D [*J*_45_]) were significantly better than those reported by Rao et al.^20^ (±0.80 D [*J*_0_] and ±1.00 D [*J*_45_]), or Ciuffreda and Rosenfield^6^ (±0.86 D [*J*_0_] and ±0.37 D [*J*_45_]), using two different commercially available portable wavefront autorefractors.

The 95% LOAs obtained in the intrasession repeatability analysis of the device were smaller than those obtained in any of the comparisons (i.e., SR1-SR2, SR1-QSFree and SR2-QSFree) for *M*, *J*_0_ and *J*_45_. These results are consistent with previous studies showing that Hartmann–Shack-based autorefraction has a higher repeatability than subjective refraction.^32,33^ Furthermore, the absence of bias found in both the repeatability and the Wilcoxon rank-sum analyses support the conclusion that the bias observed in the Bland–Altman comparisons was not caused by either the device itself or a difference in the methodology or experience of any of the five optometrists performing SR1.

No relevant clinical differences were found between VAs from SR1 and SR2. However, in 25.33% of the participants, the VAs achieved with the SR2 findings were higher than those obtained with SR1 results. This may be explained by the fact that SR2 had less time constraints and typically dedicated more effort towards optimising VAs. Conversely, optometrists performing SR1 had a higher volume of patients and consequently more time constraints.

Lastly, it is worth noting that in this preliminary assessment of the QSFree, the use of advanced algorithms, validated in prior iterations of the technology,^18,19^ fell outside of the goal of this study. It is expected that machine learning approaches, when applied to the measurement data, could further improve the agreement of the objective measurements with the subjective findings.

## CONCLUSIONS

A new portable wavefront autorefractor, the QuickSee Free, has been evaluated using an alternative protocol that considered the variability of the clinical reference standard, that is, subjective refraction. This new protocol adds to the classical study design, in which refractive findings from one device are compared to the subjective refraction performed by a single ECP, with a second subjective refraction performed by a different ECP; thus, allowing a direct estimate of the variability of the subjective refraction and intrinsically establishing a baseline to understand better the magnitude of the differences between the autorefractor and the clinical standard.

In the present study, the average difference for *M* between the QSFree and each of the two subjective refractions was found to be smaller than that between the two subjective refractions, while the astigmatic components were comparable. In contrast, the standard deviation of the differences between the device and the subjective refractions was higher than that found for the differences between the subjective refractions. Overall, the device had a high level of agreement with both subjective refractions but was found to be closer to SR2 than to SR1. These results indicate that the device can be a valuable instrument for both standard clinical examinations, as well as screening initiatives by serving as a reliable starting point for subjective refraction.

## Supplementary Information

Supplementary file (DOCX 87.7 KB)
