# Supplementary material for: Ametropia detection using a novel, compact wavefront autorefractor
Source: Ophthalmic Physiol Opt. 2023 Dec 12;44(2):311–20. doi: 10.1111/opo.13263 (PMC12872653; doi:10.1111/opo.13263)
Supplement: Supplementary file 1 — Supplementary file (DOCX 87.7 KB) [file 44402_2024_4402009_MOESM1_ESM.docx]

# Supplementary material

| 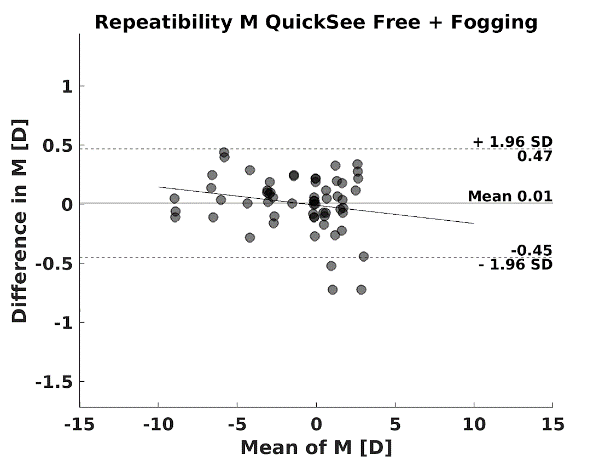 | 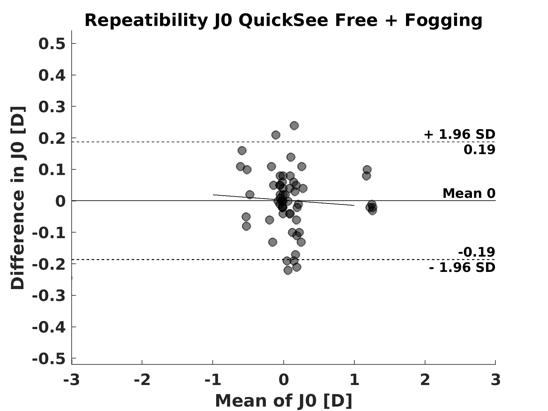 | 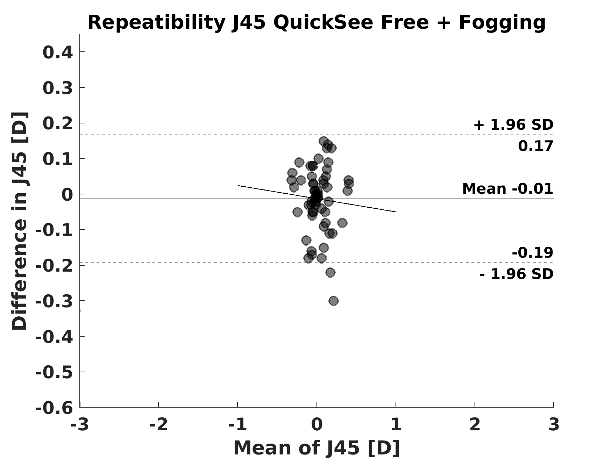 |
| --- | --- | --- |

Supplementary Figure 1. Intra-session repeatability of the QuickSee Free for M, Jo and J45 calculated on a subset of 21 subjects.

|  | **Comparison** | **Wilcoxon Rank Sum**  **M (p-value)** | **M Bias** | **M LoA** | **Wilcoxon Rank Sum**  **J0 (p-value)** | **J0 Bias** | **J0 LoA** | **Wilcoxon Rank Sum**  **J45 (p-value)** | **J_45_ Bias** | **J_45_ LoA** |
| --- | --- | --- | --- | --- | --- | --- | --- | --- | --- | --- |
| **All patients (n=75)** | **SR1-SR2** | - | -0.26 | ±0.63 | - | -0.01 | ±0.29 | - | +0.04 | ±0.26 |
|  | **SR1-QSFree** | - | +0.24 | ±1.11 | - | +0.04 | ±0.44 | - | +0.08 | ±0.36 |
|  | **SR2-QSFree** | - | -0.02 | ±1.09 | - | +0.03 | ±0.38 | - | +0.04 | ±0.33 |
| **Optometrist 1 removed (n=46)** | **SR1-SR2** | 0.81 | -0.24 | ±0.61 | 0.77 | +0.00 | ±0.31 | 0.63 | +0.06 | ±0.29 |
|  | **SR1-QSFree** | 0.76 | +0.20 | ±1.03 | 0.64 | +0.03 | ±0.44 | 0.25 | +0.10 | ±0.39 |
|  | **SR2-QSFree** | 0.79 | -0.01 | ±0.93 | 0.62 | +0.03 | ±0.40 | 0.25 | +0.20 | ±0.36 |
| **Optometrist 2 removed (n=63)** | **SR1-SR2** | 0.35 | -0.28 | ±0.61 | 0.37 | -0.02 | ±0.29 | 0.68 | +0.04 | ±0.25 |
|  | **SR1-QSFree** | 0.29 | +0.24 | ±1.10 | 0.98 | +0.06 | ±0.45 | 0.81 | +0.10 | ±0.36 |
|  | **SR2-QSFree** | 0.38 | -0.01 | ±1.12 | 0.39 | +0.04 | ±0.35 | 0.73 | -0.06 | ±0.34 |
| **Optometrist 3 removed (n=66)** | **SR1-SR2** | 0.87 | -0.26 | ±0.65 | 1.00 | -0.01 | ±0.31 | 0.78 | +0.05 | ±0.28 |
|  | **SR1-QSFree** | 0.96 | +0.24 | ±1.17 | 0.64 | +0.06 | ±0.45 | 0.75 | +0.05 | ±0.33 |
|  | **SR2-QSFree** | 0.85 | -0.02 | ±1.14 | 0.63 | +0.05 | ±0.38 | 0.87 | +0.20 | ±0.31 |
| **Optometrist 4 removed (n=55)** | **SR1-SR2** | 0.72 | -0.27 | ±0.64 | 0.55 | -0.02 | ±0.30 | 0.74 | +0.00 | ±0.21 |
|  | **SR1-QSFree** | 0.74 | +0.28 | ±1.10 | 0.96 | +0.02 | ±0.52 | 0.88 | +0.04 | ±0.39 |
|  | **SR2-QSFree** | 0.76 | -0.04 | ±1.12 | 0.78 | -0.01 | ±0.39 | 0.86 | -0.10 | ±0.33 |
| **Optometrist 5 removed (n=70)** | **SR1-SR2** | 0.96 | -0.26 | ±0.64 | 0.72 | +0.00 | ±0.25 | 0.71 | +0.04 | ±0.26 |
|  | **SR1-QSFree** | 0.92 | +0.24 | ±1.14 | 0.88 | +0.02 | ±0.36 | 0.80 | +0.10 | ±0.35 |
|  | **SR2-QSFree** | 0.90 | -0.02 | ±1.11 | 0.91 | +0.03 | ±0.38 | 0.67 | -0.05 | ±0.32 |

Supplementary Table 1. Effect on the bias and limits of agreement between SR1-SR2, SR1-QSFree, and SR2-QSFree when randomly removing from the dataset any of the 5 optometrists performing subjective refraction 1. Wilcoxon Rank-Sum p-values > 0.05 indicate that there are no significant deviations in the distributions of differences, for each power vector, after removing the patients from one optometrist.
